# Correction to: Hsa-miR-623 suppresses tumor progression in human lung adenocarcinoma

**DOI:** 10.1038/s41419-018-0880-7

**Published:** 2018-08-06

**Authors:** Shuang Wei, Zun-yi Zhang, Sheng-ling Fu, Jun-gang Xie, Xian-sheng Liu, Yong-jian Xu, Jian-ping Zhao, Wei-ning Xiong

**Affiliations:** 1 Department of Respiratory and Critical Care Medicine, Key Laboratory of Pulmonary Diseases of Health Ministry, Key Cite of National Clinical Research Center for Respiratory Disease, Tongji Hospital, Tongji Medical College Huazhong University of Science and Technology, 1095 Jie Fang Avenue, 430030 Wuhan, China; 2 Department of Surgery, Tongji Hospital, Tongji Medical College Huazhong University of Science and Technology, 1095 Jie Fang Da Dao, 430030 Wuhan, China

**Correction to**: *Cell Death Disease* (2016) **7**, e2388; 10.1038/cddis.2016.260; published online 29 September 2016

Following publication of their article, the authors noticed that there were minor errors in Figs. 3 and  7. The errors had no effect on the scientific content or conclusions. The rectified figures are given below.

**Fig. 3:**
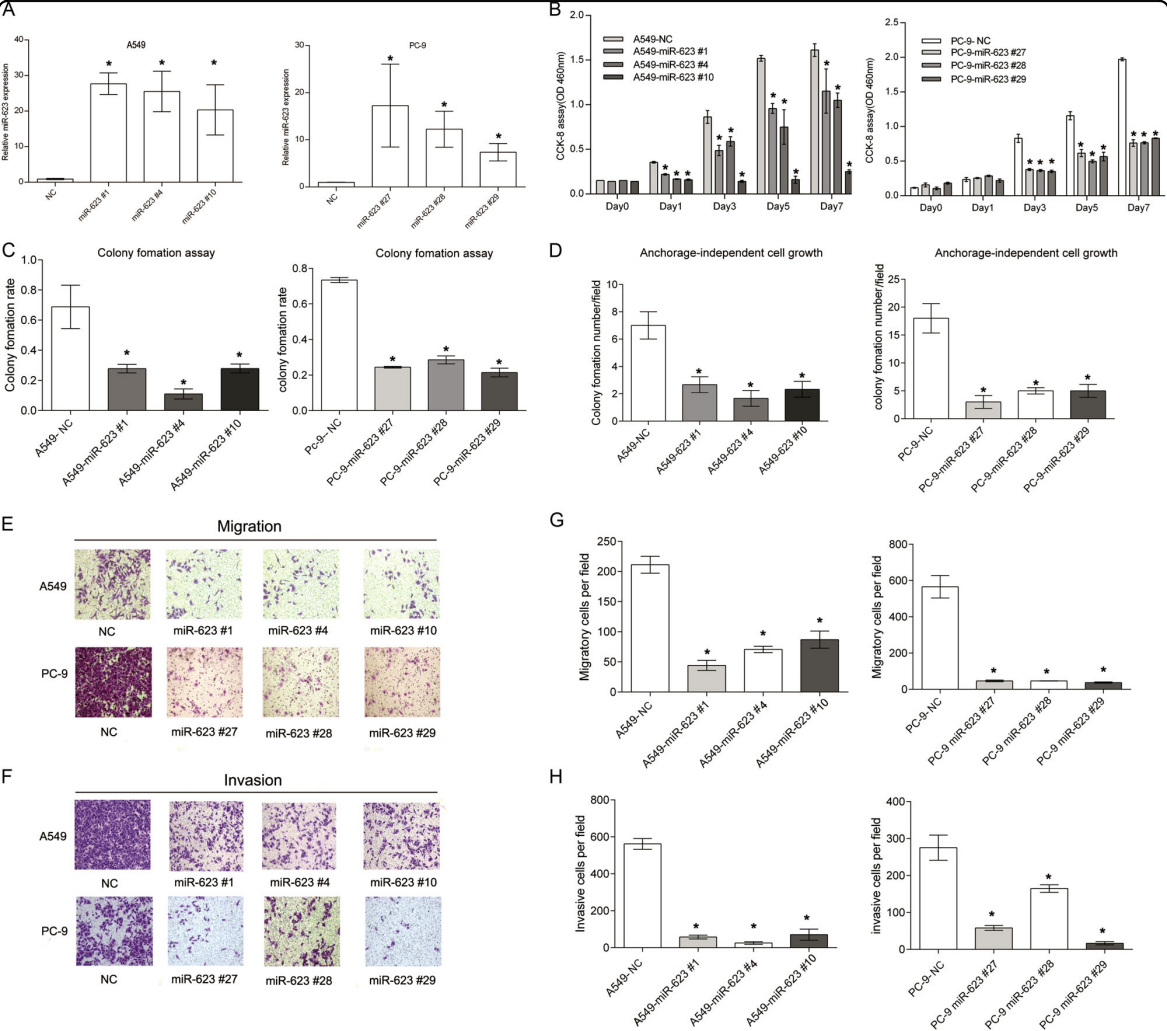
▓

**Fig. 7:**
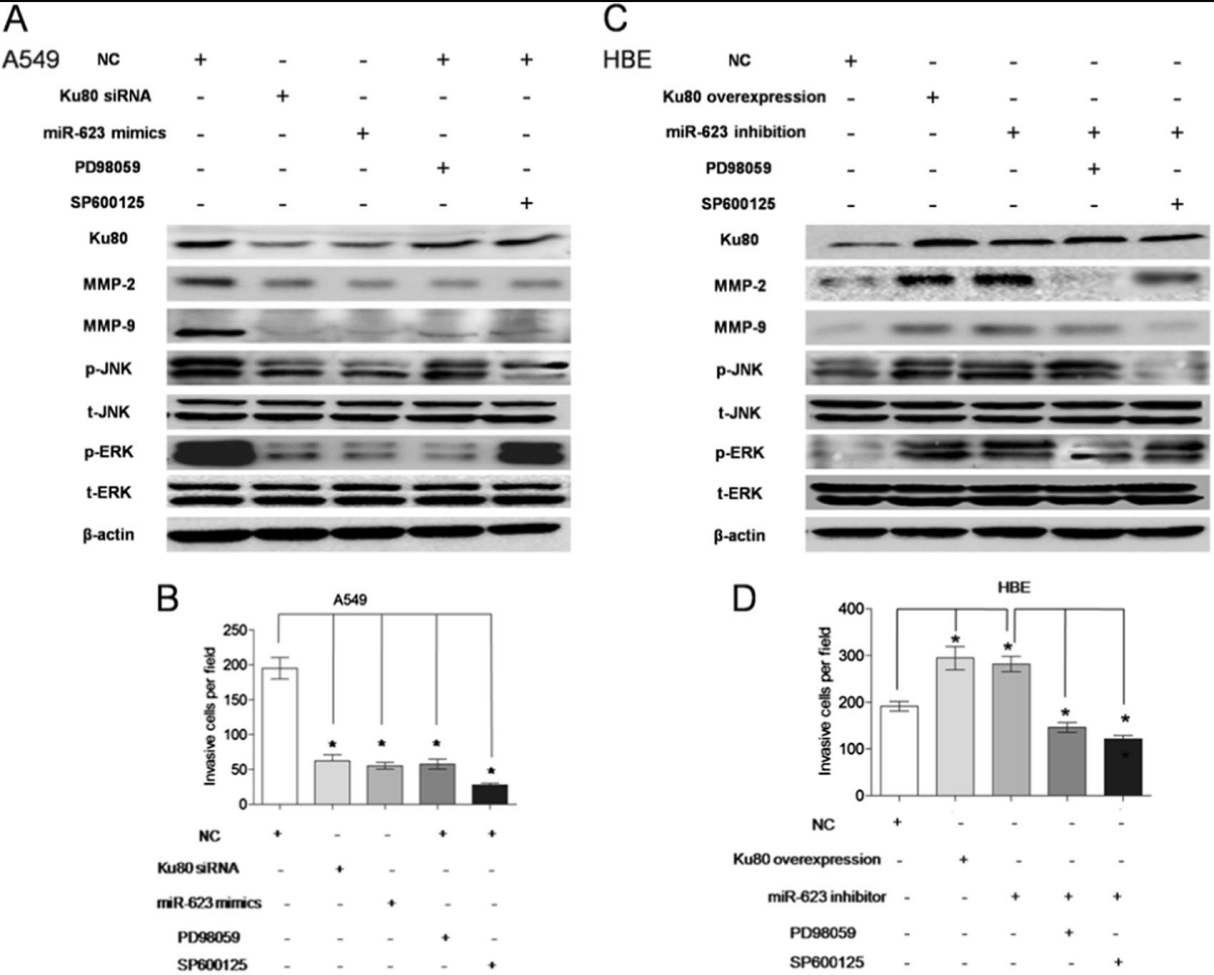
▓

The authors apologise for any inconvenience this may have caused.

